# Lipid Droplet Accumulation Promotes RPE Dysfunction

**DOI:** 10.3390/ijms23031790

**Published:** 2022-02-04

**Authors:** Tomohiro Yako, Wataru Otsu, Shinsuke Nakamura, Masamitsu Shimazawa, Hideaki Hara

**Affiliations:** 1 Molecular Pharmacology, Department of Biofunctional Evaluation, Gifu Pharmaceutical University, 1-25-4 Daigaku-nishi, Gifu 501-1196, Japan; yako.yakkou@gmail.com (T.Y.); nakamuras@gifu-pu.ac.jp (S.N.); hidehara@gifu-pu.ac.jp (H.H.); 2 Department of Biomedical Research Laboratory, Gifu Pharmaceutical University, 1-25-4 Daigaku-nishi, Gifu 501-1196, Japan; otsu-wa@gifu-pu.ac.jp; 3 Laboratory of Collaborative Research for Innovative Drug Discovery, Gifu Pharmaceutical University, 1-25-4 Daigaku-nishi, Gifu 501-1196, Japan

**Keywords:** lipid droplet, retinal pigment epithelium, phagocytosis, aging

## Abstract

Non-exudative age-related macular degeneration (AMD) is an irreversibly progressive retinal degenerative disease characterized by dysfunction and loss of retinal pigment epithelium (RPE). It has been suggested that impaired phagocytosis of the RPE is involved in the progression of non-exudative AMD, but the mechanism is not fully clear. In this study, we investigated the effect of lipid droplet accumulation on RPE function. Compared to young mice, the expression of lipid droplet-associated proteins increased in the RPE-choroidal complex, and lipid droplet in the RPE was observed in aged pigmented mice (12-month-old). Repeated treatment of the photoreceptor outer segment against ARPE-19 resulted in lipid droplets in ARPE-19 cells in vitro. Oleic acid treatment for ARPE-19 cells to form intracellular lipid droplet reduced the POS uptake into the ARPE-19 cells without causing a decrease in cell viability. The suppression of the POS uptake by lipid droplet formation improved by inhibiting lipid droplet formation using triacsin C. Moreover, the amount of intracellular reactive oxygen species was suppressed by the triacsin C treatment. These results indicate that lipid droplet is involved in the RPE dysfunction, and inhibiting lipid droplet formation may be a target for preventing and treating non-exudative AMD.

## 1. Introduction

Photoreceptor cells are essential for light reception and vision maintenance, and they receive nutritional and functional support from retinal pigment epithelium (RPE) cells. In addition, RPE maintains the retinoid cycle by phagocyting and metabolizing the photoreceptor outer segment (POS), which is excised after photoreception [1]. The non-exudative age-related macular degeneration (AMD) is a disease in which the central lesion is atrophy and loss of function of the RPE. Non-exudative AMD is classified into early AMD and advanced AMD based on its degree of progression [2]. In early AMD, lipofuscin and lipid-derived components called drusen accumulate inner or outer of the RPE cells due to dysfunction of the RPE [3,4]. Chronic accumulation of them is believed to cause cell death and geographic atrophy of RPE cells, leading to subsequent photoreceptor cell death [5,6,7]. The progression of non-exudative AMD is considered irreversible, and it is essential to intervene as early as possible. It has been reported that autophagy failure of the RPE is involved in the development and progression of non-exudative AMD [3,4]. After the RPE phagocyte of the POS, it metabolizes by a mechanism called LC3-associated phagocytosis and maintains the visual cycle [1]. It is also known that POS digestion disrupts the visual cycle and reduces visual function, indicating that phagocytosis plays an essential role in maintaining visual function [1]. It is known that phagocytosis activity is reduced in RPE cells derived from the elderly and non-exudative AMD patients [8]. Clarifying the mechanism of reduced phagocytosis activity of RPE is essential for establishing prevention and treatment strategies for non-exudative AMD.

Lipids and fatty acids play an important role in the progression of AMD, as evidenced by the fact that a high-fat diet is one of the risk factors for AMD [9]. When linoleic acid, a type of unsaturated fatty acid, is ingested, vascular endothelial growth factor levels are increased in the eyes of mice, and when experimental choroidal angiogenesis is performed, an expansion of the area of neovascularization is observed [10]. Furthermore, the increased concentration of malondialdehyde and deposition of lipid-derived components in the tissues of patients with age-related macular degeneration, and the observation of autofluorescence in the lesions, clearly indicate that lipids are a factor involved in the progression of AMD [10,11,12,13]. Previous reports have shown that accumulation of lipofuscin and lipids in the RPE precedes and causes cell death of the RPE, as in early AMD [2]. We hypothesized that lipid accumulation could affect RPE function in ways other than cell death. In this study, we focused on lipid droplet, which is involved in accumulating lipids and fatty acids. Lipid droplet is organelle discovered in adipocyte cells and are related to the accumulation of lipids or fatty acids [14]. Recent reports have also shown that lipid droplet exists in non-adipocyte cells, and its role includes lipid accumulation, autophagy, and cellular senescence [15,16]. Recently, it has been reported that the accumulation of lipid droplet in microglia, which are responsible for removing waste products in the brain, reduces their phagocytosis [17]. Therefore, we expected that the same phenomenon would occur with lipid droplet accumulation in the RPE.

In this study, we focused on aging as one of the risk factors for non-exudative AMD, clarified the localization of lipid droplet in aged mice’s eyes, and investigated lipid droplet involvement in RPE.

## 2. Results

### 2.1. Lipid Droplets Were Observed in Aged Mice RPE

**Figure 1:**
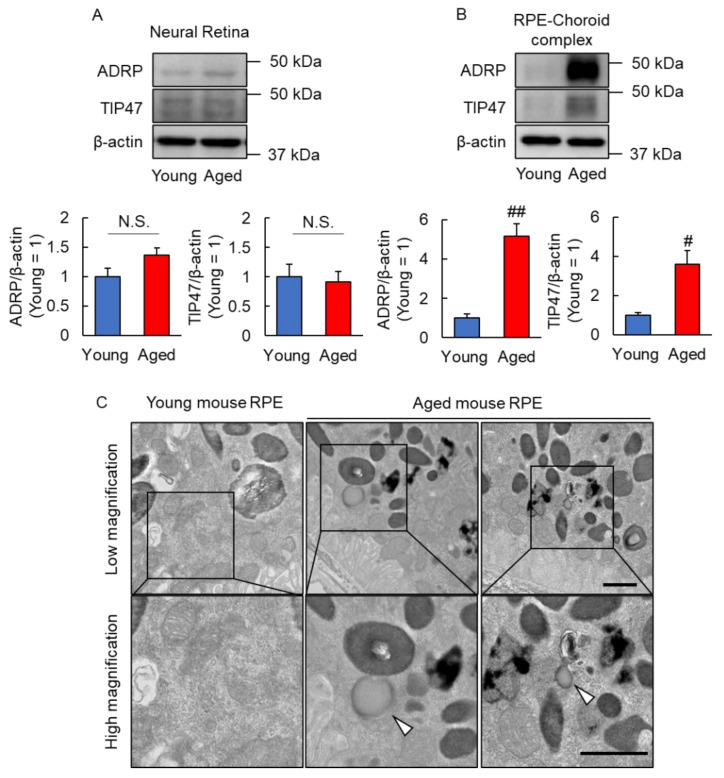
Changes in lipid droplet in the eye with age. (**A**,**B**) Expression levels of lipid droplet membrane proteins for young and aged mice in neural retina (**A**) and RPE-choroid complex (**B**). Data are the means ± standard error of means (SEMs) (*n* = 4 or 5 mice/group). ^##^ *p* < 0.01, ^#^ *p* < 0.05 vs. young (Student’s *t*-test or Welch’s *t*-test). N.S.—No significant difference. (**C**) The representative transmission electron microscope (TEM) image of the RPE cell (*n* = 3 mice/group). Arrow head shows lipid droplet. Scale bar shows 1 µm.

First, we investigated lipid droplet localization in the eyes of aged mice. We compared adipose differentiation-related protein (ADRP) and tail-interacting 47 kDa protein (TIP47), which are localized on the lipid droplet membrane [18], in the neural retina and RPE-choroid complex of young and aged mice. These protein levels did not change between young and aged mice in the neural retina (Figure 1A). On the other hand, ADRP and TIP47 expression levels increased in the RPE-choroid complex of the aged mice (Figure 1B). To visualize the presence of lipid droplet in RPE, we used transmission electron microscopy (TEM). Lipid droplet were not observed in the RPE of young mice; however, they were observed in the RPE of aged mice (Figure 1C). These results indicated that lipid droplet was formed in RPE with aging.

### 2.2. Continuous Photoreceptor Outer Segment (POS) Phagocytosis Caused Intracellular Lipid Droplet Accumulation

**Figure 2:**
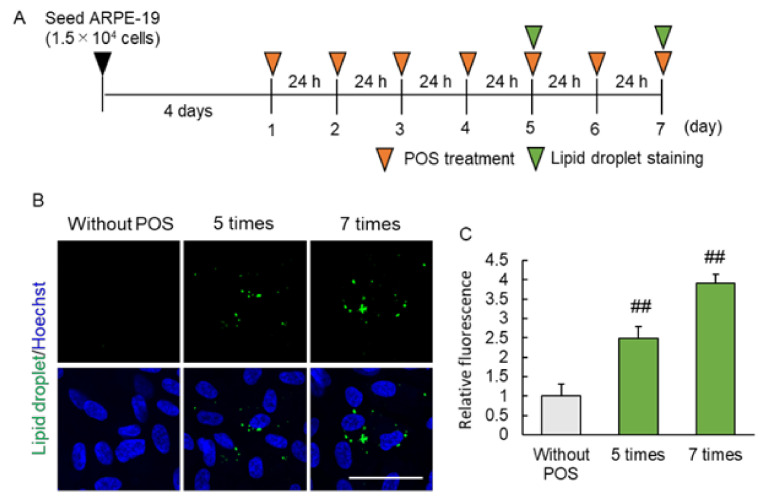
Lipid droplet accumulation via POS phagocytosis. (**A**) Experimental protocol of POS treatment induced lipid droplet accumulation. Green arrow head shows lipid droplet staining timepoint, and orange arrow head shows POS treatment timepoint. (**B**) The representative image of lipid droplet (green) and Hoechst 33,342 (blue) following continuous (5- and 7-times) POS treatment. Scale bar shows 50 µm. (**C**) The quantitative data of the fluorescence intensity of lipid droplet. Data are the means ± SEMs (*n* = 4 independent experiments). ^##^ *p* < 0.01 vs. without POS group (Dunnett’s test).

Next, we started to investigate possible mechanisms of lipid droplet accumulation by a chronic exposure of RPE cells to POS. The RPE cells are known to phagocytose the photoreceptor outer segment everyday [19], and we speculated that this may contribute to the accumulation of lipid droplet in the RPE. In this study, we investigated whether lipid droplet was formed by the continuous POS phagocytosis by RPE. While few lipid droplets were observed in absence of POS, their fluorescence intensity increased with 5- and 7-times POS treatment in a treatment frequency-dependent manner (Figure 2B,C). These results indicated that POS is the one of the causes of lipid droplet formation in the RPE.

### 2.3. Lipid Droplet Accumulation Suppressed Phagocytosis of RPE

**Figure 3:**
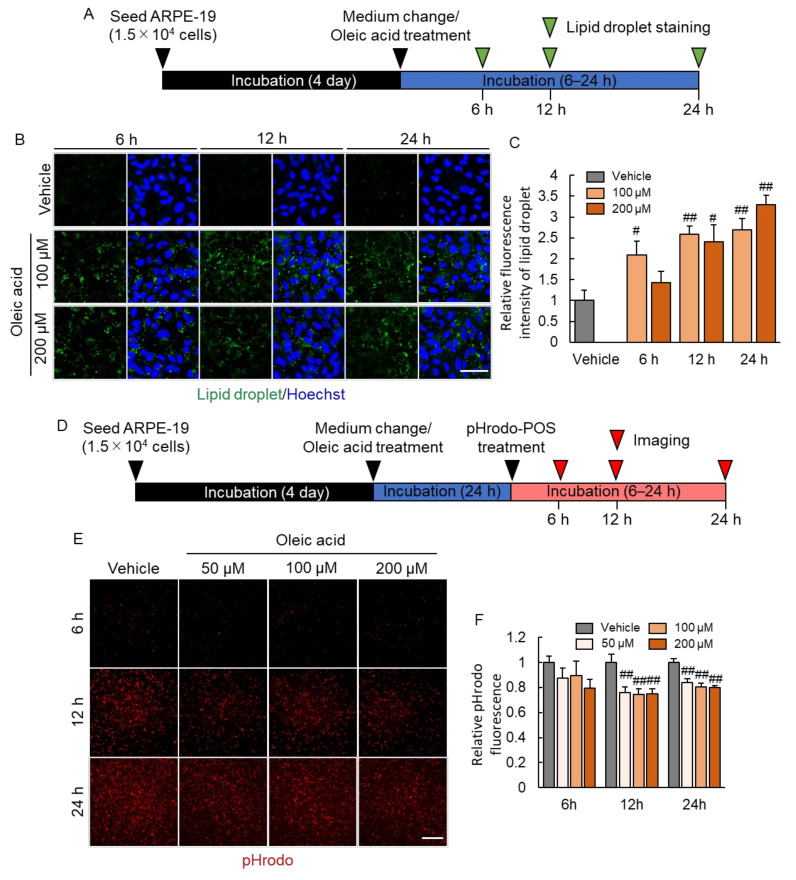
Decreased phagocytosis associated with lipid droplet accumulation. (**A**) Experimental protocol. Green arrow heads show lipid droplet staining timepoint (6, 12 and 24 h after oleic acid treatment). (**B**) The representative image of lipid droplet (green) and Hoechst 33,342 (blue) following 100 and 200 µM oleic acid treatment for 6–24 h. Scale bar shows 50 µm. (**C**) The quantitative data of fluorescence intensity of lipid droplet. Data are the means ± SEMs (*n* = 4 or 5 independent experiments). ^##^ *p* < 0.01, ^#^ *p* < 0.05 vs. vehicle (Dunnett’s test). (**D**) Experimental protocol of phagocytosis assay after oleic acid treatment. Red arrow heads show the imaging timepoint (6, 12 and 24 h after pHrodo-POS treatment). (**E**) The representative image of phagocyted pHrodo-POS in ARPE-19 cells after 50 to 200 µM oleic acid treatment. Scale bar shows 100 µm. (**F**) The quantitative data of the pHrodo fluorescence intensity. Data are the means ± SEMs (*n* = 6 independent experiments). ^##^ *p* < 0.01 vs. vehicle (Dunnett’s test).

To investigate the consequences on lipid droplet accumulation on RPE phagocytotic activity, we developed an in vitro model: we treated ARPE-19 cells with oleic acid, an unsaturated fatty acid (Figure 3A). Oleic acid treatment at concentrations of 100 and 200 µM resulted in lipid droplet formation in ARPE-19 cells (Figure 3B,C). To exclude negative effect of oleic acid on cell survival, we performed the CCK-8 assay. We found that these concentrations of oleic acid did not affect the viability of ARPE-19 cells. This model was used to evaluate the effect of lipid droplet accumulation on RPE phagocytosis. pHrodo is a pH-sensitive fluorescence dye, which is not fluorescent at neutral pH, but is activated in acidic phagosomes and to generate red fluorescence. It enables reliable detection of engulfed POS by ARPE-19 from pHrodo fluorescence. The pHrodo fluorescence intensity decreased after treatment with 100 and 200 µM oleic acid (Figure 3E,F), indicating that lipid droplet formation in RPE cell suppressed phagocytosis of POS.

### 2.4. Pharmacological Inhibition of Lipid Droplet Accumulation Improved Phagocytosis of RPE

**Figure 4:**
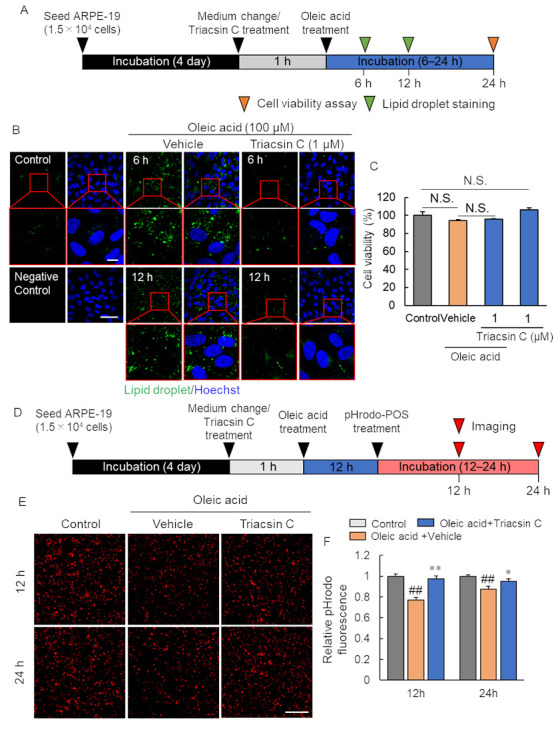
Improvement in phagocytosis by inhibition of lipid droplet accumulation. (**A**) Experimental protocol. Green arrow heads show lipid droplet staining timepoint (6 and 12 h after oleic acid treatment), and orange arrow head shows cell viability assay timepoint (24 h after oleic acid treatment). (**B**) The representative image of lipid droplet (green) and Hoechst 33342 (blue) following triacsin C and oleic acid treatment. Scale bar shows 10 µm (magnified image) or 50 µm. (**C**) The quantitative data of cell viability after triacsin C and oleic acid treatment. (**D**) Experimental protocol of phagocytosis assay after triacsin C and oleic acid treatment. Red arrow heads show the imaging timepoint (12 and 24 h after pHrodo-POS treatment). (**E**) The representative image of phagocyted pHrodo-POS in ARPE-19 cells after triacsin C and oleic acid treatment. Scale bar shows 100 µm. (**F**) The quantitative data of the pHrodo fluorescence intensity. Data are the means ± SEMs (*n* = 6 independent experiments). ^##^ *p* < 0.01 vs. control, ** *p* < 0.01, * *p* < 0.05 vs. Oleic acid + vehicle (Tukey’s test). N.S.—No significant difference.

The accumulation of lipid droplet leads to a decrease in phagocytosis of RPE. We speculated that its inhibition would improve phagocytosis. To clarify this hypothesis, we conducted a study using the acyl-CoA synthetase inhibitor: triacsin C (Figure 4A). Oleic acid treatment-induced lipid droplet formation was suppressed using 1 µM triacsin C (Figure 4B). The oleic acid at 100 µM and triacsin C at 1 µM, these concentrations observed to inhibit the formation of lipid droplet, subsequent experiments have conducted at these conditions. Then, we examined the effect of inhibiting lipid droplet formation on cell survival. The ARPE-19 cell viability was not affected by triacsin C treatment prior to oleic acid treatment and only triacsin C treatment (Figure 4C). We measured phagocytosis of RPE cells under the suppression of lipid droplet accumulation by triacsin C (Figure 4D–F). The decrease in POS phagocytosis by ARPE-19 associated with oleic acid treatment was suppressed by triacsin C treatment (Figure 4E,F). These results indicated that the suppression of lipid droplet formation in RPE improves POS phagocytosis by RPE.

### 2.5. Suppression of Lipid Droplet Accumulation Inhibited Reactive Oxygen Species Production

**Figure 5:**
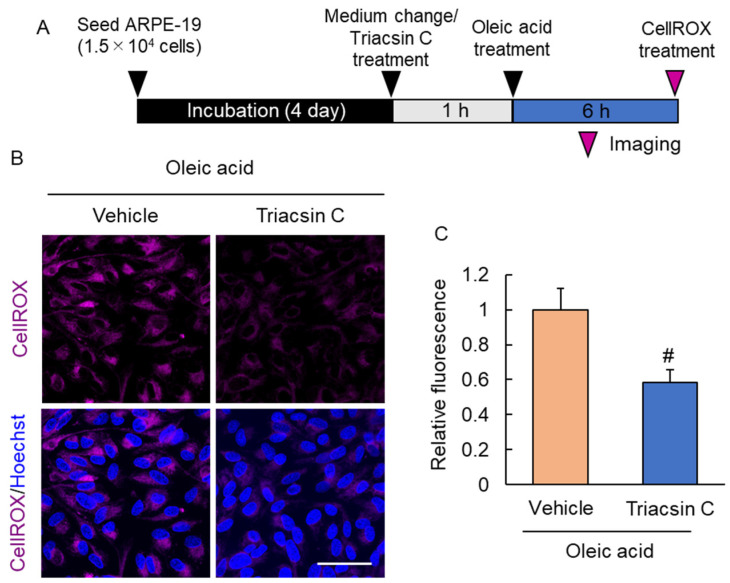
Suppression of ROS production by inhibition of lipid droplet accumulation. (**A**) Experimental protocol. Magenda arrow head shows CellROX staining timepoint. (**B**) The representative image of CellROX (magenta) and Hoechst 33342 (blue) after oleic acid and triacsin C treatment. Scale bar shows 50 µm. (**C**) The quantitative data of the CellROX intensity. Data are the means ± SEMs (*n* = 4 independent experiments). ^#^ *p* < 0.05 vs. vehicle (Welch’s *t*-test).

Reactive oxygen species (ROS) are a source of cell damage. The production of ROS in the eye leads to the development and progression of several retinal diseases. We investigated the effect of lipid droplet accumulation on intracellular ROS production. Triacsin C treatment decreased the fluorescence intensity of CellROX, suggesting that inhibition of lipid droplet formation suppresses ROS production (Figure 5).

## 3. Discussion

Lipid droplets are mainly stored in adipocytes; recent studies have reported it as an organelle in several cells [14,20]. Lipid droplets are commonly known to be involved in the accumulation of fatty acids, lipids, and having several other functions [15,16]. In this study, we investigated the role of lipid droplets in RPE function.

Aging is a risk factor for retinal degenerative diseases, such as AMD. The underlying mechanism of AMD progression is not fully understood [21]. In the past, we reported that a decrease in visual function without photoreceptor damage was observed in aged mice, and that the mechanism involved a decrease in rhodopsin expression [22]. Rhodopsin is one of the visual pigments of the retinoid cycle, and its decreased expression suggests that the retinoid cycle and phagocytosis are insufficient. The clarification of the phenotype observed in aged mice is expected to lead to the understanding of pathology of non-exudative AMD. Firstly, we aimed to determine the changes that occur in the RPE cells of aged mice. Lipid droplet formed in the RPE of aged mice, suggesting the involvement, POS uptake and fatty acid as a mechanism. Lipid-derived components such as lipofuscin and drusen have been observed in patients with AMD and are linked with the onset and progression of AMD [10,11,12,13]. Lipofuscin is a granular substance that accumulates in RPE cells over time [23]. It is an age-related byproduct of the steady phagocytosis of POS by RPE, to prevent photoreceptor damage. Drusen accumulates between the RPE and choroid, damaging the RPE cells [23]. The accumulation of these age-related changes is thought to play a role in AMD development. The accumulation of lipid droplet in the RPE of aging mice in this study may have been caused by increased lipid content in the RPE and its storage. Next, we examined the mechanism of lipid droplet accumulation. Phagocytosis of POS is the role of RPE. After receiving light, photoreceptor cells shed the POS at the ends of the photoreceptor cell, which is then engulfed by RPE every day. The phagocytized POS is degraded and synthesized by the autophagy pathway and reincorporated into the retinoid cycle [1]. The all-*trans* retinol produced in retinoid cycle has been reported to be involved in lipid droplet formation [24]. Aging is an environment in which the retinal pigment epithelium takes up POS over a long period of time. Phagocytosis of photoreceptor outer segments causes chronic all-*trans* retinol loading to RPE cells, which may lead to enhanced lipid droplet formation and expression of lipid droplet membrane proteins in aged mice. It is also likely that fatty acids were involved in the formation of lipid droplets. The POS are composed of lipids consisting of proteins, phospholipids, and cholesterol [25,26]. Phospholipids have fatty acids in their structure, which may be the source of the fatty acids. In the in vitro study, a lipid droplet was formed in the RPE when oleic acid was treated. It is known that fatty acids including docosahexaenoic acid, arachidonic acid, and oleic acid, which was used in this study, are present in the human retina [27,28]. These may have been the source that led to the formation of lipid droplets in the RPE.

The accumulation of lipid droplets has both positive and negative effects. For example, immune cells with lipid droplets, such as foam macrophages in atherosclerosis, are harmful [29]; whereas, lipid droplets are essential in supporting immune responses [30]. Whether lipid droplet accumulated cells are beneficial or detrimental may depend on factors such as environmental context or cell type. In recent years, the importance of lipids in non-exudative AMD has received increasing attention due to their accumulation in patient tissues, their association with a high-fat diet based on epidemiological studies, and genetic polymorphisms associated with lipid metabolism [31,32,33,34]. In fact, it has been reported that lipofuscin accumulation, a lipid-derived component, causes RPE cell death and consequent loss of visual function via induction of phototoxicity and inflammation [35,36,37,38]. These findings suggest that the accumulation of lipid droplet in RPE may also have a negative effect. To investigate how the accumulation of lipid droplets in the RPE affects its function, we established and evaluated an in vitro lipid droplet accumulation model. The accumulation of lipid droplets in the RPE leads to a decrease in phagocytosis; however, pharmacological inhibition of their formation improves phagocytosis. Moreover, inhibition of lipid droplet formation suppressed intracellular ROS production. Previous studies have reported that lipid accumulation is a characteristic lesion of AMD, causing the RPE cell death and subsequent loss of visual function [39]. However, since lipid accumulation is a phenomenon that occurs earlier than RPE damage, we speculated that it affects more than just RPE damage. Therefore, in this study, we focused on the phagocytic capacity of RPE. Decreased phagocytosis of RPE reduces engulfment and degradation of waste products and POS generated in the eye. The phagocytosis of RPE cells derived from donors without ocular diseases declines with age; however, it is faster in RPE derived from AMD patients [8]. It is speculated that clarifying the mechanism of decrease in phagocytosis with aging will lead to the prevention and treatment of AMD. In atherosclerotic lesions, the phagocytic activity of foam macrophages, which contain more lipid droplets, is reduced compared to macrophages that do not contain lipid droplets [40]. Microglia, which have phagocytosis as well as RPE, accumulate lipid droplets in the brain with progressing age, and inhibition of accumulation of lipid droplets enhances their phagocytosis [17]. These reports indicate that lipid droplet accumulation is related to phagocytosis. The same phenomenon was observed in RPE, suggesting that lipid droplets are a factor in the age-related decline of phagocytosis. Moreover, oxidative stress plays a major role in AMD progression. The impairment and loss of function of RPE cells associated with the generation of oxidative stress are believed to be involved in the progression of the disease [41,42]. Oxidative stress generated in the RPE is thought to cause damage to intracellular organelles and to enhance the progression of the non-exudative AMD by impairing the RPE [43]. In addition, recent reports indicate that oxidative stress reduces the RPE phagocytosis [44,45]. In other words, it is important to inhibit the generation of oxidative stress to protect the RPE from functional impairment and cellular damage. Some studies suggest that lipid droplet accumulation is enhanced by oxidative stress [46], while others suggest that lipid droplets may produce oxidative stress [17,47]. Our results suggest the latter. Since CellROX can detect many sources of oxidative stress (hydroxyl radicals, hydrogen peroxide, superoxide anion, etc.) that have been implicated in non-exudative AMD [42], the present results suggest that lipid droplet accumulation causes oxidative stress and RPE damage. However, it is not clear which type of oxidative stress is caused by the accumulation of lipid droplet, and further studies are needed. These results indicate that lipid droplet accumulation may be involved in the pathogenesis of AMD by decreasing the phagocytosis of RPE or causing oxidative stress.

There are two limitations in this study. Firstly, that it is unclear which mechanism the lipid droplets use to cause the decrease in phagocytosis of RPE. Lipid droplets are usually degraded by enzymatic degradation and β-oxidation metabolism. However, they are also degraded by lipophagy, which is a lipid droplet-selective autophagy [48,49]. The autophagy pathway is involved in the degradation of POS incorporated into the RPE by phagocytosis [1,50]. The accumulation of substances to be degraded in the cells and the load placed on them caused degradation by autophagy to become insufficient, resulting in the decrease in phagocytosis. Moreover, autophagy failure is known to occur with aging [51]. Therefore, it is possible that the decrease in phagocytosis was caused by the dysfunction of the degradation process in the RPE, and further investigation is needed to understand the mechanism. Secondly, the effects on other than phagocytosis are unknown. One of the important roles of RPE is the formation of an outer-blood–retinal barrier, which maintain homeostasis in the eye by forming tight junctions between cells and restricting the movement of substances in the intercellular spaces. In the present study, we were not able to examine the change of tight junctions and morphological changes in the RPE after lipid droplet accumulation. Future studies on the effects of lipid droplet accumulation on the structure of the RPE are needed.

In conclusion, we showed that lipid droplet accumulation arises in aged mouse RPE, and lipid droplet accumulation suppressed phagocytosis by RPE and improved ROS production. Our findings shed light on the control of lipid droplet as a novel therapeutic target for the prevention and treatment of AMD.

## 4. Materials and Methods

### 4.1. Animal

All animal experiments were approved and monitored by the Institutional Animal Care and Use Committee of Gifu Pharmaceutical University (approval number. 2017-047 [1 May 2017–31 August 2017] and 2019-128 [7 August 2020–17 March 2019]) and were performed using the Statement on the Use of Animals in Ophthalmic and Vision Research from the Association for Research in Vision and Ophthalmology. We used 2-month-old (young) and 12-month-old (aged) male C57BL/6J mice (Japan SLC, Hamamatsu, Japan). A total of 16 male mice were used in this experiment, 5 in each group for Western blotting analysis and 3 in each group for transmission electron microscope (TEM) image acquisition. They were kept under specific pathogen-free conditions of a light/dark cycle (12 h/12 h) and they consumed water and CLEA rodent diet CE-2 food (CLEA Japan, Inc., Tokyo, Japan) ad libitum. All efforts were made to minimize animal suffering.

### 4.2. Western Blotting Assay In Vivo

The eyes were enucleated from mice after euthanasia, and the retinas and RPE-choroid complex were separated from the eyes. Two eyes from one mouse were combined to make a sample, prepared samples for each mouse independently (no pooling) and used each for Western blotting assay. They were lysed using radioimmunoprecipitation assay buffer containing protease inhibitors (Sigma-Aldrich, St. Louis, MO, USA) and phosphatase inhibitor cocktail 2 and 3 (Sigma-Aldrich). The lysates followed homogenization were centrifuged for 20 min at 12,000× *g* at 4 °C. The supernatants were collected and protein concentrations were determined using the Pierce™ BCA Protein Assay Kit (Thermo Fisher Scientific, Waltham, MA, USA). After measuring the protein concentration, the lysates were mixed with a sample buffer solution containing 3-Mercapto-1,2-propanediol (×4) (FUJIFILM-Wako, Osaka, Japan) and boiled at 95 °C for 5 min. The mixed samples were separated by sodium dodecyl sulfate-polyacrylamide gel electrophoresis with 5–20% gradient gel (FUJIFILM-Wako). The separated proteins were transferred onto Immobilon-P Transfer Membrane (Merck KGaA, Darmstadt, Germany) and these were incubated and blocked in the Block One-P solution (Nacalai Tesque, Kyoto, Japan) at 25 °C for 30 min. Blocked membranes were washed with 0.05% Tween 20 (Bio-Rad, Hercules, CA, USA) containing Tris-buffered saline (T-TBS) and incubated overnight with the primary antibody at 4 °C. The membranes were washed with T-TBS and incubated with the secondary antibody at 25 °C for 2 h. Visualization of immunoreactive protein bands used ImmunoStar LD (FUJIFILM-Wako). Imaging and analysis were performed using Amersham Imager 680 (Cytiva, Tokyo, Japan). The primary antibodies used were: Adipose differentiation-related protein (ADRP) (B-6) antibody (1:500, Santa Cruz Biotechnology, Dallas, TX, USA), Tail-interacting 47kDa protein (TIP47) (F-10) antibody (1:500, Santa Cruz Biotechnology), and β-actin antibody (1:2000; Sigma-Aldrich). The secondary antibody used was horseradish peroxidase-conjugated goat anti-mouse IgG (1:2000, Thermo Fisher Scientific). β-actin was used as a loading control. The intensities of the bands for each protein were quantified and corrected for β-actin. The mean value for each group was calculated and compared relative to the value for young mice.

### 4.3. Transmission Electron Microscope (TEM) Image

The eyes were enucleated from mice after euthanasia (*n* = 3 mice/group). The eyes were fixed in phosphate-buffered 2% glutaraldehyde (Electron Microscopy Science, Hatfield, PA, USA) overnight. Subsequently post-fixed using 2% osmium tetra-oxide (Heraeus Chemicals South Africa, Port Elizabeth, South Africa) for 3 h in the ice bath. The fixed samples were then dehydrated using graded ethanol (Nacalai Tesque) and embedded using epoxy resin (TAAB Laboratories, Aldermaston, UK). Ultra-thin sections stained with uranyl acetate for 10 min and lead staining solution for 5 min were subjected to TEM observation (HITACHI H-7600). Images were taken at a distance of 500 µm from the optic nerve, and 20 images were acquired from each mouse.

### 4.4. Cell Culture

The RPE cell line, ARPE-19 cells (CRL-2302), was purchased from the American Type Culture Collection (ATCC^®^: Manassas, VA, USA) and used in this study. ARPE-19 cells culture used 10% fetal bovine serum (FBS: Biosera, Kansas City, MO, USA) contained Dulbecco’s modified Eagle’s medium (DMEM)/Ham’s F-12 (FUJIFILM-Wako), containing 100 μg/mL streptomycin (Meiji, Tokyo, Japan) and 100 U/mL penicillin (Meiji) as the antibiotic. ARPE-19 cells were passaged every 4 days by trypsinization, and these were cultured in an incubator humidified atmosphere containing 5% CO_2_ at 37 °C. In all experiments, ARPE-19 cells with passage numbers 5–9 were used. The cells were seeded at a density of 1.5 × 10^5^ cell/mL in an 8-well chamber or 96-well plate depending on the experiment. All experiments were performed after the cells were seeded and cultured for 4 days to ensure that the cells reached confluence.

### 4.5. POS Isolation from Porcine Eye and Treatment of the ARPE-19 Cell

POS isolation were acted according to previous report [22]. Briefly, retinas were isolated from fresh porcine eyes and shaken in a POS buffer containing 0.6 M sucrose. The porcine retina contained and homogenized suspension was filtered thrice using clean gauze to obtain a POS solution. The POS solution was layered gently on a linear continuous sucrose gradient (0.9, 1.2 and 1.5 M sucrose). It was centrifuged using an ultracentrifuge at 106,000× *g* for 50 min at 4 °C, subsequently the POS containing layer was collected and diluted with 5-fold ice-cold 20 mM Tris-acetate (pH 7.2) solution containing taurine (5 mM). The collected POS solution was then centrifuged at 3000× *g* for 10 min at 4 °C. After discarding the supernatant, the pellet was resuspended in 10 mL of 20 mM Tris-acetate (pH 7.2) solution containing 10% sucrose and 5 mM taurine, followed by centrifugation at 3000× *g* for 10 min at 4 °C. The pellet was resuspended in 15 mL of 20 mM sodium phosphate (pH 7.2) solution containing 10% sucrose and 5 mM taurine, followed by centrifugation at 3000× *g* for 10 min at 4 °C. The pellet was resuspended in antibiotic-free DMEM (Nacalai Tesque) and POS was counted and adjusted to 1.0 × 10^7^ POS/mL. The POS contained DMEM was stored at −80 °C. The POS solvent was thawed on ice for 2 h and warmed to 37 °C using a waterbus for 5 min. After being warmed, it was centrifuged at 2300× *g* for 5 min and the pellet was resuspended in 1 mL PBS, and the cells were treated at a concentration of 1.0 × 10^7^ POS/mL.

ARPE-19 cells were seeded in an 8-well chamber at a density of 3.75 × 10^4^ cell/well and cultured in an incubator humidified atmosphere containing 5% CO_2_ at 37 °C for 4 days. After incubation, 1 h before POS treatment, the medium was replaced with 10% FBS supplemented medium. Medium change and POS treatment was performed every 24 h, and 5 and 7 times after POS treatment, lipid droplet staining were performed following method.

### 4.6. POS Labeling by pHrodo™ Indicator

POS labeling were acted according to previous report [22,52]. We suspended the POS (2 mg/mL) in 5 mL of DMEM and mixed it with 1 mg of pHrodo succinimidyl ester (Thermo Fisher Scientific; excitation/emission = 560/585 nm) using a rotary shaker for 1 h at 4 °C. The pHrodo™ labeled-POS (pHrodo-POS) contained solution was centrifuged using an Amicon Ultra-15 (molecular weight cutoff: 3000 centrifugal filter devices; Millipore, Billerica, MA, USA) at 3000× *g* for 6 h at 4 °C. After being centrifuged, it was dispensed and stored in shading at −80 °C.

### 4.7. Lipid Droplet Staining

Lipid droplet staining was performed using Lipi-Green (Dojindo, Kumamoto, Japan) with a modification in the product protocol. After the POS or oleic acid (Nacalai Tesque) solvent treatment, the cells were washed twice with serum-free medium and incubated with a serum-free medium containing Lipi-Green at a final concentration 0.5 µM for 30 min at 37 °C. After incubation, the cells were washed twice with PBS and fixed in serum-free medium containing 3.7% formaldehyde for 15 min at 25 °C. Subsequently, the nuclei were stained using Hoechst 33342 (Thermo Fisher Scientific). The confocal images were captured using a FLUOVIEW FV3000 (Olympus) and the fluorescence intensities were measured using ImageJ/Fiji software (ver. 1.52p). Ten images were taken for each well, and their fluorescence intensity was quantified using ImageJ/Fiji software (ver. 1.52p).

### 4.8. Oleic Acid-Induced Lipid Droplet Accumulation Model In Vitro

Oleic acid treatment was performed by modifying previously reported methods [53]. Bovine serum albumin (Nacalai Tesque) was dissolved in 0.1 mol/L Tris-HCl (pH 8.0: Nacalai Tesque) at a concentration of 0.14 mg/mL. Oleic acid was dissolved in this solvent at a concentration of 4 mM and shaken gently using a rotary shaker for 6 h. Then, the solvent was filtered using a 0.22 µm filter and stored at 4 °C.

ARPE-19 cells were seeded in an 8-well chamber at a density of 3.75 × 10^4^ cell/well and cultured in an incubator humidified atmosphere containing 5% CO_2_ at 37 °C for 4 days. Four days later, the medium was changed to 10% FBS-containing medium and treated with various concentrations of oleic acid. After 6-, 12- and 24-h incubation with oleic acid, lipid droplet staining was performed according to the above method. Triacsin C (Cayman Chemical, Ann Arbor, MI, USA) was treated 1 h before oleic acid treatment at a final concentration of 1 µM and 6- and 12-h after incubation, lipid droplet staining was performed.

### 4.9. Cell Counting Kit-8 (CCK-8) Assay

Cell viability analysis was performed using a Cell Counting Kit-8 (CCK-8: Dojindo, Kumamoto, Japan). ARPE-19 cells were seeded in 96-well plate at a density of 1.5 × 10^4^ cell/well and cultured in an incubator humidified atmosphere containing 5% CO_2_ at 37 °C for 4 days. After 4 days of incubation, they were treated with various concentrations of oleic acid and incubate for 24 h. Then, the cells were washed twice with PBS and incubated with 10% FBS-containing medium. Subsequently, we added CCK-8, and cell count was measured at 450 nm using a Varioskan Flash 2.4 microplate reader (Thermo Fisher Scientific). This measurement was carried out immediately after addition and after 1 h of incubation.

### 4.10. Phagocytosis Assay Using pHrodo-POS

ARPE-19 cells were seeded in 96-well plates at a density of 1.5 × 10^4^ cell/well and incubated for 4 days. Oleic acid was added after the medium was changed. Triacsin C was treated 1 h before oleic acid treatment. Twelve or twenty-four hours after incubation with oleic acid, the medium was changed to DMEM/Ham’s F-12 containing 10% FBS. One hour after incubation, 1 × 10^5^ pHrodo-POS/well was treated to every well and cultured for 6 to 24 h. The extracellular POS were washed five times using DMEM/F12 containing 10% FBS at each time point. Six wells were used in each group, and one image was taken using a BZ-X700 all-in-one fluorescence microscope (Keyence, Osaka, Japan) for each well, and its fluorescence intensity was quantified using ImageJ/Fiji software (ver. 1.52p).

### 4.11. Intracellular Reactive Oxygen Species (ROS) Detection

Intracellular ROS were detected using CellROX^®^ Deep Red (Thermo Fisher Scientific) following the manufacturer’s staining protocol. ARPE-19 cells were seeded in an 8-well chamber at a density of 3.75 × 10^4^ cell/well and cultured in an incubator humidified atmosphere containing 5% CO_2_ at 37 °C for 4 days. Four days later, the medium was changed to 10% FBS-containing medium and treated with oleic acid. Triacsin C was treated 1 h before oleic acid treatment. Six hours after oleic acid treatment, CellROX^®^ reagent diluted in PBS at a final concentration of 5 μM was added to the cells and incubated for 30 min at 37 °C. Subsequently, the cells were counterstained with Hoechst 33342 and fixed with 3.7% formaldehyde (FUJIFILM-Wako) for 15 min. Samples were imaged using FLUOVIEW FV3000 (Olympus) within 2 h. Ten images were taken for each well, and their fluorescence intensity was quantified using ImageJ/Fiji software (ver. 1.52p), and the mean value was used for quantitative data.

### 4.12. Statistical Analyses

Statistical analyses were performed using the SPSS statistical software version 24.0.0.0 (IBM, Armonk, NY, USA). Bar charts depict the mean ± standard error of mean, as indicated in each figure legend. Statistical analyses of the groups were performed as indicated in the figure legends. Equality of variance was confirmed using Levene’s test. Means between two groups were compared by the two-tailed Student’s *t*-test or Welch’s *t*-test. Multiple groups comparison was analyzed one-way analysis of variance (ANOVA), followed by Dunnett’s or Tukey’s post hoc multiple comparisons test. Statistical significance was set at *p* < 0.05.

## Data Availability

Data is contained within the article.
